# A novel kefir product (PFT) inhibits Ehrlich ascites carcinoma in mice via induction of apoptosis and immunomodulation*

**DOI:** 10.1186/s12906-020-02901-y

**Published:** 2020-04-28

**Authors:** Nariman K. Badr El-Din, Sameh M. Shabana, Bashar A. Abdulmajeed, Mamdooh Ghoneum

**Affiliations:** 1 Department of Zoology, Faculty of Science, University of Mansoura, Mansoura, 35516 Egypt; 2 Department of Surgery, Charles R. Drew University of Medicine and Science, Los Angeles, California USA

**Keywords:** *Lactobacillus kefiri*, PFT, Apoptosis, Cell cycle, T cells

## Abstract

**Background:**

The popularity of fermented foods such as kefir, kuniss, and tofu has been greatly increasing over the past several decades, and the ability of probiotic bacteria to exert anticancer effects has recently become the focus of research. While we have recently demonstrated the ability of the novel kefir product PFT (Probiotics Fermentation Technology) to exert anticancer effects in vitro, here we demonstrate its ability to inhibit Ehrlich ascites carcinoma (EAC) in mice.

**Methods:**

Mice were inoculated intramuscularly with EAC cells to develop solid tumors. PFT was administered orally (2 g/kg/day) to mice 6 days/week, either 2 days before tumor cell inoculation or 9 days after inoculation to mice bearing solid tumors. Tumor growth, blood lymphocyte levels, cell cycle progression, apoptosis, apoptotic regulator expression, TNF-α expression, changes in mitochondrial membrane potential (MMP), PCNA, and CD4+ and CD8+ T cells in tumor cells were quantitatively evaluated by flow cytometry or RT-PCR. Further studies in vitro were carried out where EAC cells along with several other human cancer cell lines were cultured in the presence of PFT (0–5 mg/mL). Percent cell viability and IC_50_ was estimated by MTT assay.

**Results:**

Our data shows that PFT exerts the following: 1) inhibition of tumor incidence and tumor growth; 2) inhibition of cellular proliferation via a marked decrease in the expression of tumor marker PCNA; 3) arrest of the tumor cell cycle in the sub-G0/G1 phase, signifying apoptosis; 4) induction of apoptosis in cancer cells via a mitochondrial-dependent pathway as indicated by the up-regulation of p53 expression, increased Bax/Bcl-2 ratio, decrease in the polarization of MMP, and caspase-3 activation; and 5) immunomodulation with an increase in the number of infiltrating CD4^+^ and CD8^+^ T cells and an enhancement of TNF-α expression within the tumor.

**Conclusions:**

PFT reduces tumor incidence and tumor growth in mice with EAC by inducing apoptosis in EAC cells via the mitochondrial-dependent pathway, suppressing cancer cell proliferation, and stimulating the immune system. PFT may be a useful agent for cancer prevention.

## Background

Cancer remains the largest cause of mortality in the world*.* Cancer develops from the uncontrolled growth of a proliferating cellular clone due to acquisition of self-sufficiency in growth signals, insensitivity to anti-growth signals, the ability to evade apoptosis, and limitless replicative potential [1]. Conventional treatments for cancer, such as chemotherapy, can be effective, but these drugs have high toxicity and can lower patients’ quality of life. Thus, there is an urgent need to develop alternative treatments with fewer side effects that can improve patient health.

One of the most promising current developments for treatment is actually a method that has been used to improve health for over 100 years: the consumption of probiotic products containing lactic acid bacteria (LAB). LAB is composed of a group of bacteria that degrade carbohydrates (e.g., via fermentation) with the production of lactic acid. Over a century ago, Metchnikoff acknowledged that the regular consumption of LAB in fermented dairy products such as yogurt was associated with enhanced health and longevity [2]. Probiotics have been used as therapies for digestive health for over a century, and their potentially beneficial effects on bacterial flora in the body have led to an increasing number of studies of probiotics and/or *Lactobacillus* strains on digestive and gynecological pathologies. A probiotic therapy (VSL#3) has been shown to be effective against pouchitis [3–5], a probiotic preparation containing *Bifidobacterium infantis* has been shown to reduce irritable bowel syndrome symptoms [6], *S. boulardii* and LABs significantly decreased the incidence of antibiotic-associated diarrhea [7–9], and many *Lactobacillus* strains hold promise for treating bacterial vaginosis [10] and recurrent urinary tract infections [11]. Recent studies have also revealed that kefir, a LAB-rich fermented milk drink made from kefir grains, can have several positive bioactivities, including antioxidant, antimicrobial, anti-inflammatory, and healing activities [12, 13], as well as improvement of bone mass in an ovariectomized rat model of postmenopausal osteoporosis [14].

More significantly, increasing evidence has been mounting of the anticancer effects of LAB in many in vivo, in vitro, and epidemiological studies [15–24]. Such studies have shown probiotics to be effective against many cancers such as colorectal [18], intestinal [19], colonic/rectal [20], oral [21], and breast cancer [22, 23]. Epidemiological studies have found an inverse correlation in humans between the frequency of yogurt consumption and the risk of breast cancer, indicating that probiotic bacteria might reduce the risk of cancer in humans [24]. One potentially beneficial probiotic product is PFT (Probiotics Fermentation Technology). PFT is a novel kefir grain product composed predominantly of LAB strains: ~ 90% *Lactobacillus kefiri P-IF* along with 2–3% of another *L. kefiri* compound and three yeast strains [25, 26]. PFT has already been shown to exert anticancer effects in vitro against multidrug-resistant (MDR) human myeloid leukemia cells (HL60/AR) cells [26] and human gastric cancer cells [27]. This data is in support of other work that has also shown Lactobacillus strains to have effects in vitro against bladder [28] and gastric cancer [29], as well as inhibitory effects in animals with breast [22, 23, 30], intestinal [19], colon [20], and oral cancer [21] and in humans with colon [31], liver [32], and breast cancer [24].

These results motivated this study’s investigation of PFT’s action in vivo and its exploration of PFT’s mechanisms of action. LAB has been demonstrated to exert anticancer effects through several different mechanisms, including the inhibition of potential pathogens and carcinogenesis in the gut by binding to and degrading carcinogens, enhancement of antioxidant activities, production of antitumorigenic or antimutagenic compounds, and enhancement of the host’s immune response [33, 34]. Probiotics have also been shown to induce apoptosis in many different cancer cell lines such as monocytic leukemia-cell line THP-1 [35], chronic myeloid leukemia-derived cells [36], and colon cancer cell line SNUC2A [37].

In this study, we aimed to evaluate the anticancer effect of PFT against animal bearing Ehrlich ascites carcinoma (EAC) and to investigate the potential mechanisms of action. Furthermore, we supported our study of EAC in mice with a study of PFT’s action against EAC in vitro, along with several other human cancer cell lines.

## Methods

### Probiotics Fermentation Technology (PFT) kefir grain product

PFT is a mixture that contains primarily (~ 90%) a heat-killed freeze-dried form of *L. kefiri* P-IF. PFT also consists of ~ 2–3% of the following: one bacterial strain *L. kefiri* P-B1, and yeast strains *Kazachstania turicensis*, *Kazachstania unispora* and *Kluyveromyces marxianus*. P-IF is a specific LAB strain with a unique DNA sequence, and PET scans show a 99.6% homology with regular kefiries. The characteristics of P-IF have been reported [25, 26]; the exact chemical composition is under active investigation. The yeast strains are not intentionally added, but rather are present in large amounts when obtaining the product from the Caucasus mountains and are filtered out in order to maximize the kefiri levels. PFT was provided by Paitos Co., Ltd., Yokohama, Kanagawa, Japan.

### Preparation of EAC cells and tumor transplantation

EAC is a well-established murine model used over the last four decades for studying breast cancer. It is originally hyperdiploid and an undifferentiated carcinoma with unique characteristics such as 100% malignancy, short life span, high transplantable capability, and rapid proliferation [38–41]. In this study, murine EAC cells were obtained from the National Cancer Institute, Cairo University, Egypt. Cells were maintained in vivo in female Swiss albino mice via weekly intraperitoneal passage of cells. Mice were inoculated intramuscularly in the right thigh of each mouse with 0.2 ml of EAC containing 2.5 × 10^6^ viable cells in PBS to develop solid tumors. Tumor cell viability was found to be 95%, as examined by Trypan blue dye exclusion method.

### Preparation of human cancer cell lines

The current study used three human tumor cell lines for in vitro study: liver carcinoma cell line (HepG2), breast carcinoma cell line (MCF-7), and colon carcinoma cell line (CACO-2). Tumor cells were purchased from American Tissue and Culture Collection, Manassas, VA, USA. These cells were maintained in a complete medium consisting of RPMI-1640 that was supplemented by 10% fetal calf serum, 2 mM glutamine, and a mixture of 100 μg/ml streptomycin and penicillin. Cells were permitted to grow in tissue culture flasks (Corning, USA) and were incubated at 37 °C in a humidified atmosphere of 5% CO_2_ and 95% air.

### Animals

Sixty-nine female Swiss albino mice were purchased for this study from the National Cancer Institute, Cairo University, Egypt. They weighed between 19 and 21 g and were 2 months old. Mice were kept in alternating 12-h light and dark cycles at constant temperature (24 ± 2 °C) and 10% relative humidity. They were given water and standard cube pellets ad libitum. Pellets consisted of wheat flour (80%), casein (12.5%), bran (3.3%), olive oil 300 (2.3%), fats (1.0%), DL-methionine (0.5%), vitamins and salt mixture (0.2%), and water (0.2%). The total calorie breakdown was 9% fat, 73% carbohydrate, and 18% protein. Pellets were purchased from Misr Oil & Soap Company, Cairo, Egypt. All animal protocols were followed in compliance with the Guide for the Care and Use of Laboratory Animals at the University of Mansoura, Egypt, and the study was approved by the Committee on the Ethics of Animal Experiments of the University of Mansoura, Egypt, on January 4, 2015.

### Experimental design

**Fig. 1:**
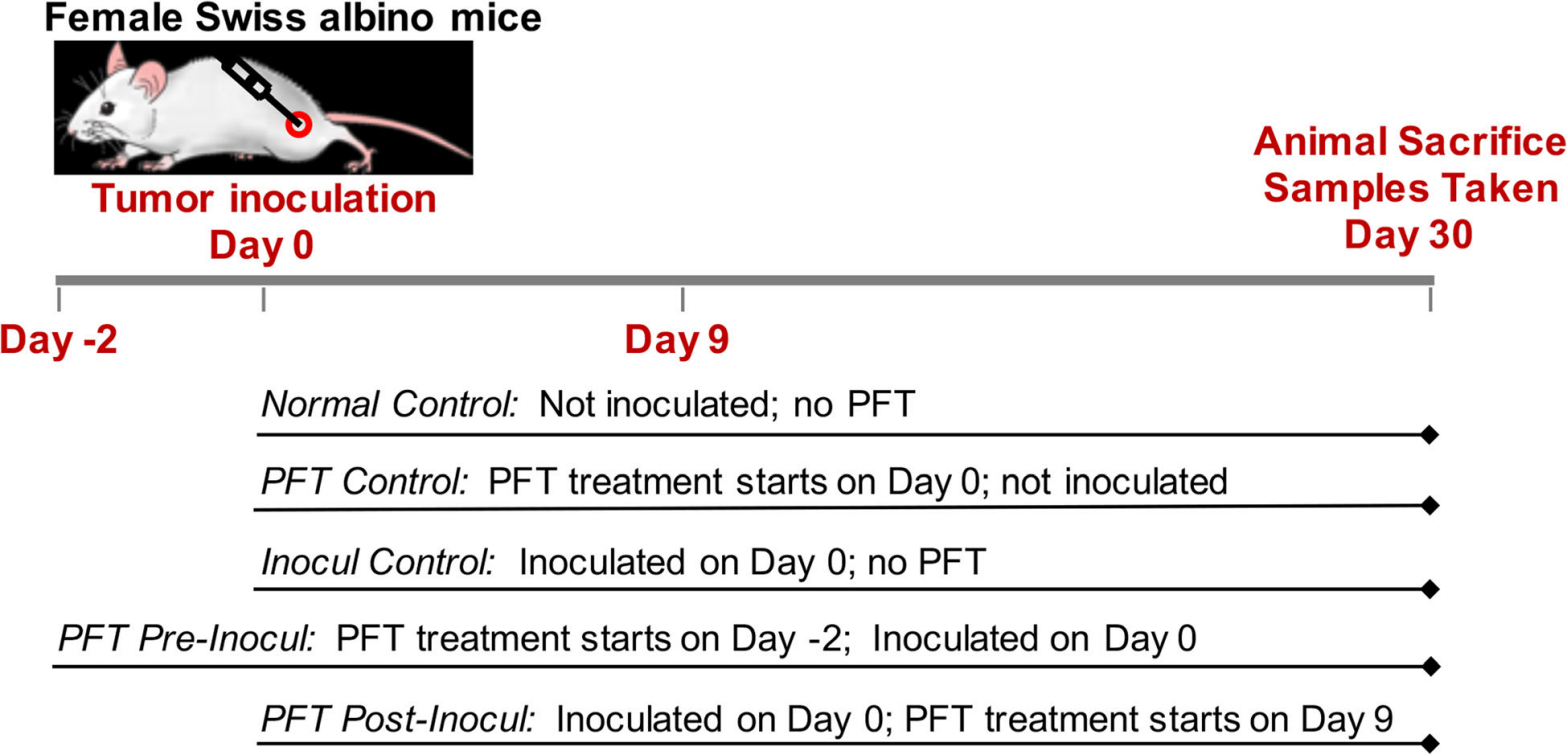
Experimental design

Mice were divided randomly into five groups: Group-1, the vehicle, (Normal Control): Mice without tumor inoculation and untreated with PFT; Group-2 (PFT Control): Control mice treated with PFT without inoculation; Group-3 (Inocul Control): Mice bearing tumor without PFT treatment; Group-4 (PFT pre-inocul): Mice treated with PFT 2 days prior to tumor inoculation, PFT treatment continued until day 30; and Group-5 (PFT post-inocul): Mice treated with PFT 9 days post tumor inoculation, PFT treatment continued throughout the experiment (30 days). PFT was administered orally six times per week over the course of the study. The dose utilized was 2 g/kg/day, based on earlier findings by others [42]. Fig. 1 illustrates the experimental design and the different treatment groups.

Parameters under investigation were as follows: tumor growth, cell cycle progression, apoptosis, apoptotic and cell cycle regulators expression, changes in mitochondrial membrane potential (MMP), PCNA, and CD4+ and CD8 + T cells in tumor cells, blood lymphocytes level, and TNF-α expression.

### Change in body weight (BW)

Mice in the five groups were assessed for changes in body weight: (initial BW on Day 0, last and net final BWs on day 30). Net final BW = (final BW – tumor weight). We determined BW gain as the difference between initial and net final BW.

### Tumor incidence and tumor growth evaluation

The potential antitumor effects of PFT was examined by checking daily for palpable tumors and measuring any changes in tumor volume (TV/mm^3^) and tumor weight (TW/g). Measurements of TV (3 days/week) was carried out via digital Vernier calipers. Measurements were taken from day 9 to day 30 post tumor cell inoculation. Data gathered was analyzed to obtain tumor volume using the following formula: TV (*mm*^3^) = 0.52*AB*^2^, where *A* and *B* are the minor and major axis, respectively. Percentages of tumor growth inhibition in mice receiving PFT were calculated. At the end of the experiment, solid tumors were excised to determine TW/g.

### Sample collections

Sample collections were drawn at day 30. Mice were then weighed and anesthetized using sodium pentobarbital (40 mg/kg BW, i.p.). Blood was drawn from the abdominal aorta using heparinized plastic syringes, before being transferred into anticoagulation test tubes in order to measure lymphocytes levels. Mice were euthanized by cervical dislocation and afterward were dissected in order to obtain solid tumor. Tumor tissues were immediately frozen in preparation for our various investigations.

### Flow cytometric analysis

#### Cell preparation for flow cytometry

Excised tumor tissues were taken from mice bearing solid Ehrlich carcinoma (SEC). Tissue samples were diced and rubbed through fine nylon gauze (40–50 mesh count/cm, HD 140 Zuricher Buteltuch fabrik AG). Afterward, samples were washed through the gauze with Tris–ethylenediaminetetraacetic acid (Tris-EDTA) buffer at pH 7.5 [0.47 g of 0.005 M Tris-EDTA; 1.022 g of 0.07 M HCl; 3.029 g of 0.1 M Tris-(hydroxymethyl aminomethane)]. Cells were subsequently suspended in PBS, centrifuged at 200–300 g for 5 min, and then resuspended in sterile PBS (cell density = 1 × 10^6^ cells/ml). Cells were then fixed and permeabilized with ice-cold 70% methanol in PBS. Until used, the cells were stored at −20 °C.

#### Cell cycle analysis by propidium iodide

Suspensions of tumor cells were centrifuged. Cell pellets were then resuspended in a 1 ml solution of propidium iodide (PI) in the dark for 30 min. Cells were subsequently examined via flow cytometry (Becton Dickinson, San Jose, CA). Data was analyzed using the MODFIT program (Verity Software House, Inc., Topsham, ME, USA) for DNA analysis. The computer software was used to calculate the coefficient of variation around the peak in G0/G1, along with each sample’s percentage of cells in each of the DNA cell cycle phases (G0/G1, S, and G2/M). If a distinct peak separate from the G1 diploid peak deviated by over 10% from the diploid internal standard, or if the G1 peak deviated from a corresponding G2/M peak more than 10%, then an aneuploid cell population was classified as present. Calculations were also performed to obtain the apoptosis index (AI)/proliferation index (PrI) ratio.

#### Detection of apoptosis by AnnexinV/PI double staining

The ability of PFT to induce apoptosis in tumor cells was identified and quantified via flow cytometry. This study used the Annexin V conjugated alexafluor 488 apoptosis detection kit (BD Biosciences, San Jose, CA); manufacturer’s instructions were followed. The study also conducted FACS analysis using Cell Quest 3.3 software. Early apoptotic cells fluoresce green when stained with Alexa488 and, on the fluorescence-activated cell sorting histogram, they show up in the lower right (LR) quadrant. Late apoptotic cells, when stained with both Alexa488 and PI, give red-green fluorescence and present in the upper right (UR) quadrant of the histogram. Necrotic dead cells, when stained with PI only, present in the upper left (UL) quadrant.

#### Effect of PFT on mitochondrial membrane potential (MMP)

MMP variations during apoptosis were examined using 3,3′-Dihexyloxacarbocyanine iodide (DOC6(3)) (Molecular Probes, Eugene, OR, USA). 5 × 10^5^ cells/ml were incubated with 0.5 mM DOC6(3) for 30 min at 37 °C. Cells were subsequently transferred onto ice for FACS analysis. Forward and side scatters were employed to gate and exclude cellular debris using a FACScan. Cells were then excited at 488 nm before green fluorescence was collected on FL1 at 530 nm. Five thousand cells were analyzed. Data was acquired and then analyzed using Cell Quest software (Becton Dickinson).

#### Expression of cell cycle progression, apoptosis and cell proliferation related protein

Mouse monoclonal antibodies against P53 (sc-7480), Bcl-2 (sc-7382), Bax (sc-7480), caspase-3 (sc-7272), p21 (sc-6246), p27 (sc-1641), PCNA (sc-56) protein, and other reagents were purchased from Santa Cruz Biotechnology, Inc., Dallas, Texas USA. Tumor cells (1 × 10^6^) taken from PFT-treated mice were incubated using the appropriate antibody for 1 h and then incubated again with FITC-conjugated goat anti-rabbit antibody. Afterwards, cells were thoroughly washed with PBS with BSA and then analyzed using a flow cytometer.

#### T helper cells (CD4+) and T cytotoxic cells (CD8+)

FACS analysis of CD4+ and CD8+ T cells infiltrating tumor tissues was performed using mouse anti-CD4+ FITC (clone GK1.5) and mouse anti-CD8+ FITC (clone 53–6.7), (BD Pharmingen, San Diego, CA**)**. Tumor cells were suspended in PBS at a concentration of 1 × 10^6^ cell/ml. Cells were prepared as described above before being centrifuged. The supernatant was discarded. Cell pellets were then re-suspended in 500 μl PBS before 1 ml of suspension was dispensed in flow cytometric tube. Cells were incubated with 25 μl of anti-CD4+ or anti-CD8 in dark for 30 min at 4 °C. After the supernatant was discarded, the cells were washed twice by PBS, pH 7.2. Two hundred microliter paraformaldehyde solution was then added to each tube, mixed well, and kept in dark at 4 °C till FACS analysis was conducted according to the manufacturer′s instructions.

### Detection of TNF-α relative gene expression by reverse transcription-polymerase chain reaction (RT-PCR)

Total RNA extraction was carried out using a GF-TR-050 Total RNA Extraction Kit (Vivantis Technologies SDN. BHD., Malaysia) according to the manufacturer’s instructions. The total RNA was reverse transcribed (RT) into cDNA by using FastQuant RT Kit (Tiangen Biotech (Beijing) Co., Ltd) in line with manufacturer’s guidelines. The kit contained gDNase, which can remove genomic DNA by incubation at 42 °C for 3 min to protect the total RNA from genomic DNA interference. Real-time RT-PCR was conducted using Maxima SYBR Green qPCR Master Mix (2X) Kit (Thermo Scientific). The reaction conditions and data analysis were conducted in compliance with the manufacturer’s instructions. 5μl of cDNA in a total volume of 25 μl containing 12.5 μl Maxima SYBR Green qPCR Master Mix (2X), Forward Primer 0.3μMol, Reverse Primer 0.3μMol (TNF-α: 5-TGAACTTCGGGGTGATCGGT-3; 5-GGTGGTTTGTGAGTGTGAGGG-3. β-actin: 5-CAGGATTCCATACCCAAGAAG-3; 5-AACCCTAAGGGCAACCGTG-3.), ROX Solution 10 nM/100nM, up to 25 μl by Water nuclease-free. Thermal cycling condition 95 °C for 10 min, followed by 40 cycle of 95 °C for 15 s, 58 °C for 30 s, 60 °C for 30 s. Reactions were run on an PIKO REAL 96 Real-Time PCR system (Thermo Scientific). TNF-α was designed by (Biolines, USA). Gene expression differences between groups were calculated using the ΔΔC Ct (cycle time, Ct) method according to Livak and Schmittgen [43]. These differences were normalized against *β-actin* and expressed as relative mRNA levels, as compared to controls.

### Evaluation of the in vitro cytotoxic effect of PFT on cancer cell lines by MTT assay

For in vitro study, we used four tumor cell lines: murine EAC, human HepG2, human MCF-7, and human CACO-2. Tumor cells (1 × 10^4^/well) were incubated with ascending concentrations of PFT (0.6, 1.25. 2.5 and 5 mg/ml) in 96-wells. The final volume of medium in each well after the addition of PFT was 200 μl. The cultures were then incubated at 37 °C, 5% CO2 with 98% relative humidity for 24 and either 48 or 72 h. Afterwards, 50 mg of MTT were added and the cultures were incubated for an additional 4 h. The plates were then centrifuged before the medium was carefully removed. The formazan crystals were then solubilized with acid alcohol and the plates were read at 590 nm by using an ELISA plate reader (Molecular Devices, Menlo Park, CA, USA). The 50% inhibitory concentration (IC50) was determined as the drug concentration resulting in a 50% reduction of cell viability. The IC50 was determined by plotting the logarithm of the drug concentration versus the survival rate of the treated cells.

### Statistical analysis

Reported data values are mean ± standard error (SE). Analysis was conducted with ANOVA (one-way analysis of variance) followed by Dunnett’s post-hoc test in order to identify the significance for multiple comparisons. Statistical significance was determined at the *p* < 0.05 level. With regard to the Results section shown later, 8 mice per group were utilized for statistical analysis for Fig. 5. However, only 6 of these mice from each group were usable for all other biochemical analyses, due to a shortage of tumor samples. The sample size of 6 was still large enough to yield statistically significant information.

## Results

Several parameters in vivo were carried out at 30 days post treatment with PFT.

### Effect of PFT on body weight

The body weight of EAC bearing mice without treatment decreased by 22.3% relative to their initial body weight (data not shown). This final body weight was significantly lower when compared against the final body weights of the normal control and PFT-treated control groups. On the other hand, treatment with PFT prevented this body weight loss due to cancer, an effect that was observed for both pre- and post-inoculation groups.

### Effect of PFT on tumor growth

**Fig. 2:**
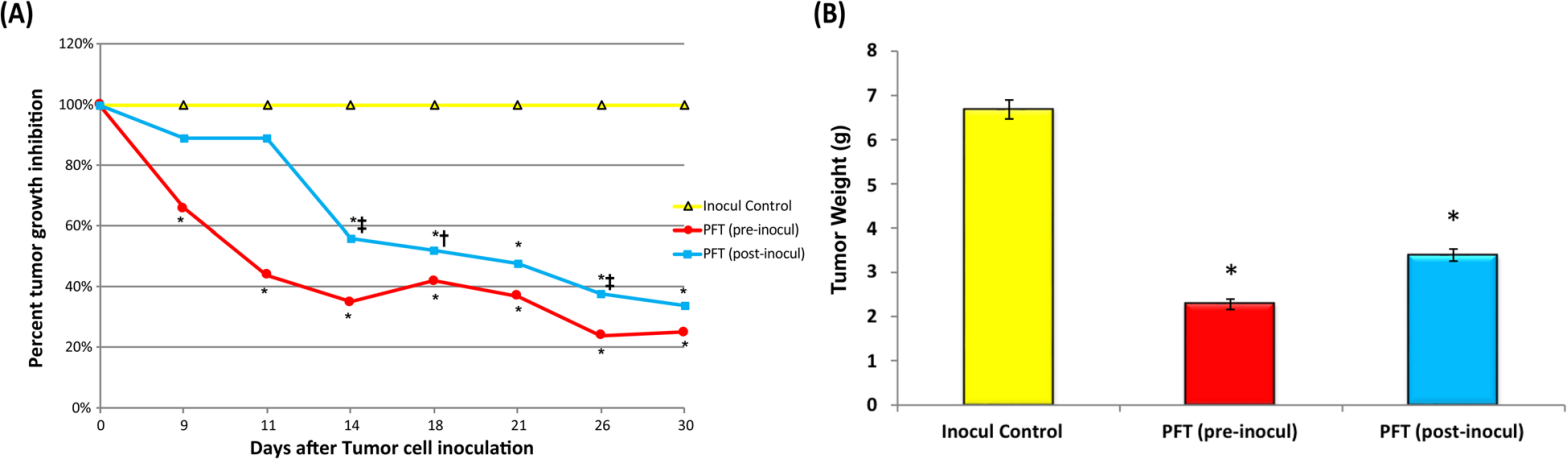
In vivo effect of PFT on tumor growth. **a**) Tumor volume (TV) was recorded at different intervals over 30 days and the percent TV inhibition is shown. Values represent the mean ± SE. Similarly, PFT was seen to decrease tumor weight. **b**) Number of mice/group: Inocul Control: (11), PFT (pre-inocul): (10), and PFT (post-inocul) (16). *, *P* ≤ 0.01 vs. inocul control group; †, *P* < 0.05 vs. pre-inocul group; ‡, *P* < 0.01 vs. pre-inocul group

Mice receiving PFT before EAC inoculation (the PFT pre-inocul group) showed tumor incidence in 76.5% (13/17) of the animals recorded on day 9, and complete tumor regression was noticed in three mice (3/13). Mice in this group also showed a significant percentage of tumor volume (TV) inhibition, detected at day 9 and maximized (75%) at day 30 (Fig. 2a), while mice that received PFT after EAC inoculation (the PFT post-inocul group) showed a significant inhibition in TV by 67% at day 30 relative to the untreated control group. Similar trends in tumor weight (TW) post-treatment with PFT were noted. Fig. 2b shows a 64.6 and 48.6% decrease in TW for pre-and post-inoculation groups, respectively (*p* < 0.01).

### Effect of PFT on cell proliferation

**Fig. 3:**
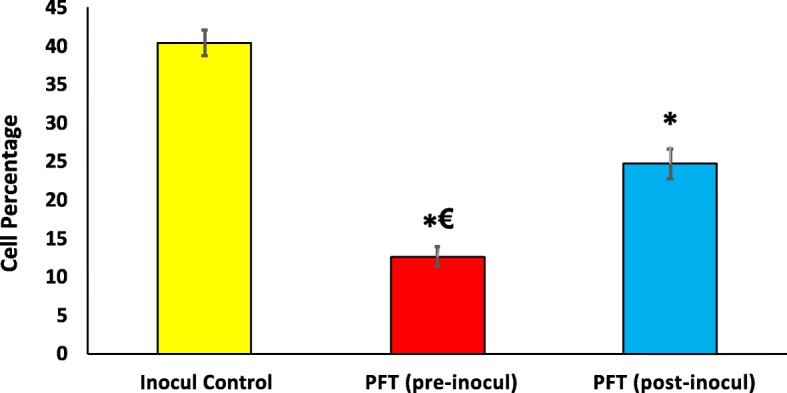
Effect of PFT treatments on PCNA expression in tumor tissues as determined by flow cytometry. Values represent the mean ± SE of 6 mice/group. *, *P* ≤ 0.01 vs. inocul control group; €, *P* < 0.01 vs. post-inocul group

The expression of proliferating cell nuclear antigen (PCNA) post-treatment with PFT was examined. As shown in Fig. 3, pretreatment with PFT resulted in a decrease of 68.7% in levels of PCNA expression, while posttreatment with PFT showed a decrease of 38.9% as compared to the inoculated control group.

### Effect of PFT on cell cycle progression

**Fig. 4:**
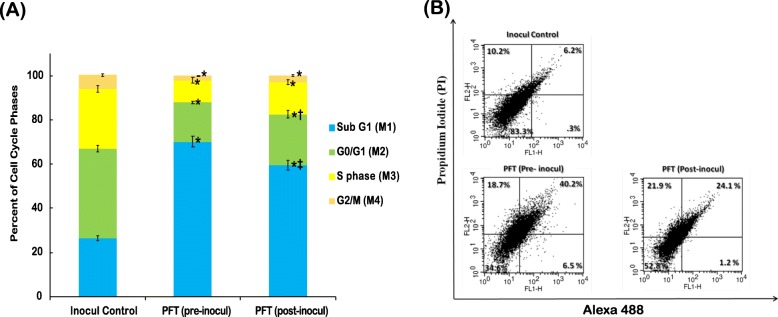
**a**) Percent change in the distribution of cell cycle phases in tumor tissues of Inocul mice treated with PFT. Each value represents the mean of 8 mice/group. * *P* ≤ 0.01 vs. inocul control group; † *P* < 0.05 vs. pre-inocul group; ‡ *P* < 0.01 vs. pre-inocul group. **b**) Effect of PFT on apoptosis as detected by Annexin V/PI and analyzed by flow cytometry in EAC bearing mice. The early apoptotic cells stained with Annexin V-conjugated Alexa flour 488 dye were shown in the lower right (LR) quadrant of the histogram, whereas late apoptotic cells were shown in the upper right (UR) quadrant. Viable cells were shown in lower left (LL), while the necrotic dead cells were in the upper left (UL) quadrant of the histogram. One representative histogram is shown from 6 individual mice/group

Data in Fig. 4a show that the percentage of sub-G1 phase hypodiploid cells were significantly increased for those groups that received PFT before (166%) or after (126%) tumor inoculation (*p* < 0.01), as compared to the inoculated control group. On the other hand, treatment with PFT caused a reduction in the percentage cell population in other phases relative to the inoculated control group, with pretreatment and posttreatment of PFT, respectively: G0/G1 (56, 43%), S (63, 46%), and G2/M (69, 56%). The effect of PFT on Apoptosis index/ Proliferation index ratio (AI/PrI) was also examined. Pretreatment with PFT increased the AI/PrI ratio by 242% while posttreatment with PFT resulted in an increase of 140% (*p* < 0.01), as compared with the inoculated control group (data not shown).

### Quantitative determination of apoptosis by AnnexinV/PI staining

Quantitative flow cytometric analysis of apoptosis was performed by AnnexinV/PI double staining. As shown in Fig. 4b, pretreatment with PFT caused a large decrease in the percentage of viable cells (− 61%, *p < 0.01)*, while posttreatment with PFT showed − 44% (*p < 0.01*) as compared to the inoculated control group. On the other hand, pretreatment and posttreatment with PFT significantly increased early apoptotic population by + 385% and + 310%, *p < 0.01*, respectively, as compared to the inoculated control group. Similar pattern of PFT treatment was shown on late apoptotic cells for pretreatment and posttreatment groups recorded + 578% and + 421%, *p < 0.01*, respectively, relative to the inoculated control group. In addition, necrotic population of pretreatment and posttreatment with PFT recorded − 65% and − 47%, *p < 0.01*, respectively, of the inoculated control group.

### Effect of PFT on cell cycle and apoptotic regulators

The percentage of cell cycle and apoptotic regulators in tumor tissues were examined (Table 1). Results showed that PFT induced apoptosis in vivo in cancer cells via the mitochondrial-dependent pathways. This was indicated by a significant increase in p53 expression by 261 and 139% for pre- and posttreatment, respectively. Besides, treatment with PFT significantly increased P21 and P27 expression as compared with the inoculated control group. The effect of PFT was higher in mice receiving PFT before tumor inoculation than mice receiving PFT post inoculation. Furthermore, results showed that Bax expression increased by 333 and 152%, Bcl2 expression decreased 45 and 29%, Bax/Bcl2 ratio increased 741 and 276%, and caspase-3 increased by 123.50 and 89.00% for pre- and posttreatment, respectively, as compared with control untreated mice.

**Table 1:** Effect of PFT on cell cycle and apoptotic regulators in tumor tissues of the different groups as determined by flow cytometry

Groups	Inocul Control	PFT (pre-inocul)	PFT (post-inocul)
Parameter			
P53 expression	17.55±0.95	63.46± 6.72^*^	42.05± 6.68^*†^
% change from inocul control	-	+261%	+139%
P21 expression	12.03± 0.92	55.29± 1.68^*^	39.60± 4.71^*‡^
% change from inocul control	-	+359%	+229%
P27 expression	23.11± 1.61	60.46± 1.52^*^	35.96± 1.37^*‡^
% change from inocul control	-	+161%	+55%
Bax expression	12.15± 0.62	52.66± 3.16^*^	30.62± 1.34^*‡^
% change from inocul control	-	+333%	+152%
Bcl2 expression	66.89±2.34	36.47±0.89^*^	47.15±1.68^*‡^
% change from inocul control	-	-45%	-29%
Bax/Bcl2 ratio	0.17±0.008	1.43±0.075^*^	0.64±0.038^*‡^
% change from inocul control	-	+741%	+276%
Caspase-3 expression	20.98±1.63	46.89±2.32^*^	39.67±0.66^*†^
% change from inocul control	-	123.50%	89.00%
MMP	71.14±3.27	15.86±3.69^*^	43.06±3.65^*†^
% change from inocul control	-	-77.7%	-39.5%

Each value represents the mean±SE of 6 mice/group. ^*^P ≤ 0.01 vs. inocul control group; ^†^*P* < 0.05 vs. pre-inocul group; ^‡^*P* < 0.01 vs. pre-inocul group

Effect of PFT treatment on the MMP was also examined. Results indicate that pre- and posttreatments with PFT caused significant decrease in mitochondrial polarization, 77.7 and 39.5%, respectively.

### Immunological effects of PFT treatment

The effect of PFT on several immunological parameters in tumor tissues was examined.

#### Percentage of CD4+ T and CD8+ T cells infiltrated in the tumor tissue

**Fig. 5:**
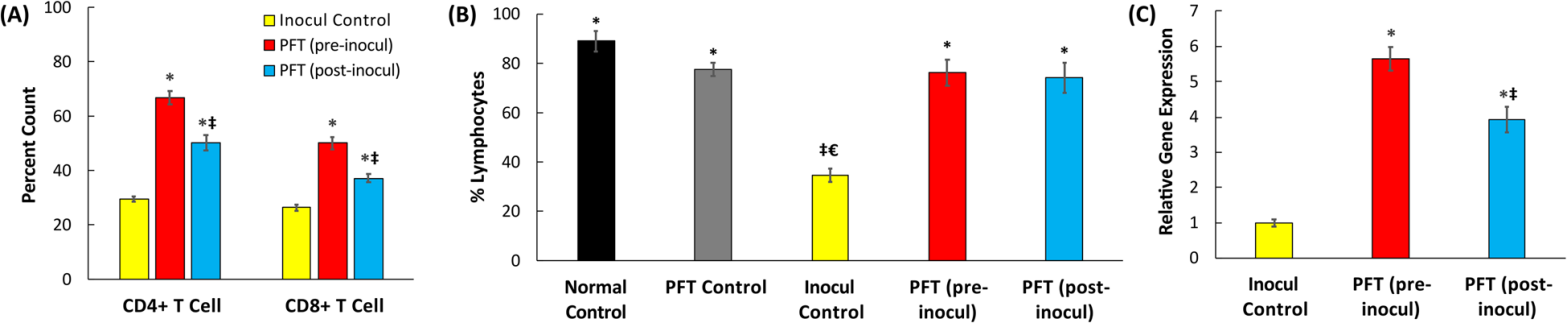
Effect of PFT treatment on immunological parameters. **a**) CD4+ and CD8+ infiltrating tumor tissue of different groups*.* Each value represents the mean ± SE of 6 mice/group. **b**) Effect of PFT on percent lymphocytes in the blood of the different groups. Each value represents the mean ± SE 6 animals/group. **c**) Effect of PFT on relative gene expression of TNF-α in tumor tissues of different groups as determined by RT–PCR. Each value represents the mean ± SE of 5 tumor samples /group. *, *P* ≤ 0.01 vs. inocul control group; ‡, *P* < 0.01 vs. pre-inocul group; €, *P* < 0.01 vs. post-inocul group

Figure 5a show that mice that received PFT treatment demonstrated a significant increase in the levels of CD4+ T cells infiltrating the tumor: a 2.3-fold and 1.7-fold increase for mice with pretreatment and posttreatment, respectively. A similar pattern of increased levels of CD8+ T cells infiltrating in the tumor tissue in mice treated with PFT was also noted but to a lower extent.

#### Percentage of lymphocytes in the blood

Results in Fig. 5b show that mice in the inoculated control group demonstrated significant decrease in the percentage of lymphocytes (60.9%), relative to normal control mice. Mice with PFT pretreatment or posttreatment maintained the percentage of lymphocytes within the values of normal control mice.

#### Relative gene expression of TNF-α

Figure 5c shows results of the effect of PFT on relative gene expression of TNF-α in tumor tissues of EAC-bearing mice as determined by RT–PCR. Treatment with PFT shows remarkable increase in the relative gene expression. Pretreatment with PFT resulted in 5.7-fold increase and posttreatment with PFT showed 3.9-fold increase in the relative gene expression as compared to the inoculated control group.

### Evaluation of the in vitro cytotoxic effect of PFT on various tumor cell lines

**Fig. 6:**
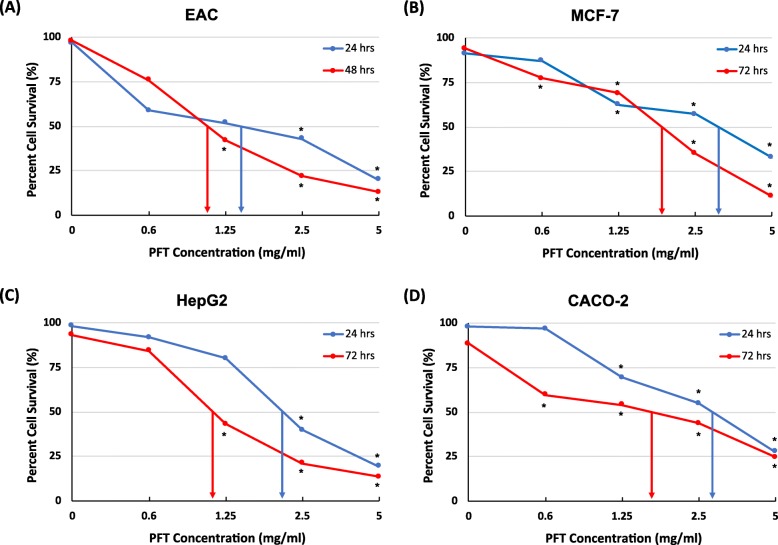
Cytotoxic effect of PFT on **a**) EAC, **b**) MCF-7, **c**) Hep-G2, and **d**) CACO-2 cell survival by MTT assay. Cancer cells were cultured in the presence of different concentrations of PFT (0.6, 1.25. 2.5 and 5 mg/ml) for 24 and either 48 or 72 h, and the IC_50_ values were determined. The figure is a representative of three independent and identical experiments. *, *P* ≤ 0.01 vs. untreated control

The in vitro cytotoxic effect of PFT on several tumor cell lines was examined by MTT assay. These cell lines included mouse EAC and three human cancer cell lines: breast cancer MCF-7, hepatocellular carcinoma HepG2, and colon cancer CACO-2. PFT induced cytotoxicity against EAC cells at 24 h and increased over time. The IC50 values at 24 and 48 h were 1.3 and 1.1 mg/ml, respectively (Fig. 6a). Similarly, PFT also exhibited cytotoxic effect against the human cancer cell lines at 24 h and increased over time. (Fig. 6b-d). Results show that there was a gradation in the sensitivity of human cancer cell lines toward the toxic effect of PFT: HepG2 > CACO-2 > MCF-7. Similar trends in results for all cell lines was detected by trypan blue assay (data not shown).

## Discussion

Results of the current study demonstrate that PFT induces chemoprotective effects against mice bearing Ehrlich ascites carcinoma (EAC). Treatment with PFT prior to tumor inoculation prevented tumor growth in 23.5% of the mice, and complete tumor regression was recorded in 23% of mice (3/13) after day 21. PFT treatment also resulted in a decrease in tumor volume, with pretreatment showing 75% decrease and posttreatment showing 67% decrease. This data is in accordance with earlier studies by others which showed that treatment with probiotics causes significant suppression in growth of multiple types of cancers in experimental animals [44]. Probiotics have been shown to be effective against colorectal and intestinal [18–20], oral [21], and breast cancer [22–24]. Most of these studies used chemically-induced tumors; however, in the current study, tumors were initiated through inoculation of Ehrlich ascites carcinoma (EAC) in mice. These studies suggest that LABs have the ability to suppress tumor growth regardless of the tumor initiators.

Our earlier in vitro studies showed that PFT probiotic exerts anticancer effect on various cancer cells via different mechanisms. For example, PFT induced apoptosis in murine metastatic breast cancer (4 T1) cells and in myeloid leukemia cells via a hole-piercing mechanism [36, 43], as well as in AGS human gastric cancer cells via decreasing the polarization of MMP and Bcl2 expression [27]. In the current study, flow cytometry study showed PFT acts as a potent apoptotic agent in EAC cells in vivo, as indicated in the sub-G1 phase by cell cycle arrest with a marked increase in the hypodiploid cell population. Furthermore, marked increases were recorded in the apoptosis index/proliferation index (AI/PrI) of 3.4- and 2.4-fold for mice supplemented with PFT prior to EAC inoculation and post EAC inoculation, respectively, relative to the inoculated control group. An earlier study also revealed that waste milk whey suppresses tumor cell proliferation by interfering with the cell cycle [45]. Analysis revealed a dramatic up-regulation in the percentage of protein levels of p53, p21, and p27 in tumor cells of animals treated with PFT. p53 has the ability to induce cell cycle arrest and apoptosis [46], while p21 and p27 bind to cyclin-CDK complexes to inhibit their catalytic activity and induce cell cycle arrest [47].

In addition, our data showed that PFT induced a modulation of apoptotic regulators, including up-regulation of p53 and Bax expression, down-regulation of Bcl2 expression, and increased Bax/Bcl2 ratio. We also noted a significant decrease in mitochondrial polarization and over 2-fold increase in caspase-3 expression posttreatment with PFT. Taken together these data suggest that PFT induces apoptosis via the mitochondrial-dependent pathway. These results are in accordance with other studies showing that other probiotics such as Propionibacterium freudenreichii [48] and conjugated linoleic acid, a functional lipid produced from *Lactobacillus plantarum*, have an apoptotic effect via the mitochondrial-dependent pathway in different cancer cell lines [49].

In the current study, the level of PCNA expression in untreated mice significantly increased. Conversely, a down-regulation of percent PCNA level was observed in mice supplemented with PFT prior to EAC inoculation and post EAC inoculation by 68.7 and 38.7%, respectively, as compared to inoculated control mice. Suppression of cellular proliferation may represent one of the mechanisms through which probiotic PFT exerts its chemopreventive effects. Similar results were noted in studies of the probiotic Dahi, produced by *L. lactis*, which showed suppressed PCNA expression in colorectal tissue of Wistar rats [50]**.**

The anticancer effect of probiotics is heavily investigated in cancers of the gastrointestinal tract (GI tract) [18–20]. However, its effect in other types of cancer is less studied. In the current study, PFT was administered orally, and its ability to exert an anticancer effect on cancers not in the GI tract is of special interest. The effects of bioactive molecules secreted by probiotics may represent another mechanism by which probiotics exert their effects. A few of these bioactive molecules have been discussed in the literature, including parasporin-2Aa1 (from *Bacillus thuringiensis* strain A1547) [51], epsilon-poly-L-lysine (from marine *Bacillus subtilis* SDNS) [52], and polyphosphate (poly P) (from *L. brevis* SBL8803) [53].

Immunomodulation may represent another important mechanism by which probiotics exert their anticancer activity. In the current study**,** supplementation with PFT for mice bearing tumor resulted in: 1) significant increase in the percentage of CD4+ T and of CD8+ T cells infiltrating tumor tissue, 2) recovery of the percentage of lymphocytes in the blood, and 3) substantial increase in the relative gene expression of TNF-α. The mechanisms by which PFT enhances the response of T cells may include the action of activated dendritic cells (DCs). Our earlier study showed the ability of PFT to activate DCs to induce CD4+ T and CD8+ T cell responses in vitro [54], with PFT-activated DCs upregulating CD103 and CD107a expression and increasing Granzyme-B’s granular content in CD8+ T cells. CD103+ CD8+ T cells have been shown to increase tumor necrosis and prevent cancer progression in mice [55], and CD107a and Granzyme-B expression by CD8+ T cells is known to be a hallmark of cytotoxic T cells that can help eliminate cancer cells [56, 57]. Other studies have also shown the potential of probiotics as immune modulators. These include the ability of LABs to enhance the number of total T cells, NK cells, MHC class II+ cells, and CD4-CD8+ T cells in mice [58] and increase the phagocytic activity of macrophages in mice bearing tumor [59]. In addition, daily intake of *L. casei Shirota* for 3 weeks significantly increased NK cell activity of cigarette smokers [60].

The data in Fig. 6 shows that PFT has an anti-cancer effect against multiple types of cell lines in vitro, including HepG2 (human liver cancer), MCF-7 (human breast cancer), and CACO-2 (human colorectal cancer). However, we noticed that there is a differential response among these cell lines toward the cytotoxic effect of PFT. These cytotoxic effects of PFT are in accordance with others who showed that probiotics promote anti-proliferative or proapoptotic activities in various human cancer cells/cell lines, including colonic and gastric cancer cells [37, 61, 62], blood cancer cells such as chronic myeloid leukemia-derived and monocytic leukemia cells [27, 35, 36], breast cancer cells [63], and cervical cancer cells [64].

Cachexia has been shown to be a major cause of mortality and morbidity in cancer patients [65] and resulting weight loss can affect the quality of life of patients with advanced cancer [66]. Animal studies have shown that as soon as the tumor is palpable, adipose tissue wasting can occur [67]. In the current study, mice bearing tumor showed a significant decrease in body weight, as compared to normal control. However, treatment with PFT prevented body weight loss due to cancer. This data is in accordance with earlier studies that have shown other probiotics can similarly help maintain body weight in the presence of cancer [68, 69].

The current definition of probiotics associates them with live cells, and therefore viability is considered to be a fundamental property of probiotics. However, the current study, along with the work of others, shows that heat-killed probiotics have the ability to generate beneficial biological responses [70]. For example, dietary supplementation of cell-wall preparation of *Enterococcus faecalis* strain EC-12 exerts an immunostimulatory effect in chicks [71], heat-killed *Enterococcus faecalis* FK-23 preparation (FK-23) stimulates the non-specific immune responses in healthy dogs [72], and heat-killed bifidobacteria enhances cytokine production in clonal murine macrophage and Tcell lines [73]. In addition, the effectiveness of both viable probiotics [74] and heat-killed *L. acidophilus* LB [75] have been reported for the treatment of diarrhea. These studies suggest that probiotics induce their effects in both alive and heat-killed forms.

## Conclusions

We conclude that *Lactobacillus kefiri* PFT may have chemopreventive potential to reduce tumor incidence and tumor growth by inducing apoptosis in EAC cells via the mitochondrial-dependent pathway, suppressing cancer cell proliferation, and stimulating the immune system. These results may suggest the applicability of PFT for cancer prevention and/or treatment in clinical trials.

## Data Availability

The datasets used and/or analysed during the current study are available from the corresponding author on reasonable request.

## Abbreviations

AI: Apoptosis index
BW: Body weight
DOC6(3): 3,3′-Dihexyloxacarbocyanine iodide
EAC: Ehrlich ascites carcinoma
LAB: Lactic acid bacteria
LR: Lower right
MDR: Multidrug-resistant
MMP: Mitochondrial membrane potential
PCNA: Proliferating cell nuclear antigen
PI: Propidium iodide
PrI: Proliferation index
RT: Reverse transcribed
RT-PCR: Reverse transcription-polymerase chain reaction
SE: Standard error
SEC: Solid Ehrlich carcinoma
Tris-EDTA: Tris–ethylenediaminetetraacetic acid
TV: Tumor volume
TW: Tumor weight
UR: Upper right
UL: Upper left
